# The role of GABA in modulation of taste signaling within the taste bud

**DOI:** 10.1007/s00424-024-03007-x

**Published:** 2024-08-29

**Authors:** Ayaka Mikami, Hai Huang, Aiko Hyodo, Kengo Horie, Keiko Yasumatsu, Yuzo Ninomiya, Yoshihiro Mitoh, Seiji Iida, Ryusuke Yoshida

**Affiliations:** 1 Department of Oral Physiology, Graduate School of Medicine, Dentistry and Pharmaceutical Sciences, Okayama University, Okayama, Japan; 2 Department of Oral and Maxillofacial Reconstructive Surgery, Graduate School of Medicine, Dentistry and Pharmaceutical Sciences, Okayama University, Okayama, Japan; 3 Faculty of Medicine, Dentistry and Pharmaceutical Sciences, Okayama University, 2-5-1, Shikata-Cho, Kita-Ku, Okayama, 700-8525 Japan; 4 Tokyo Dental Junior College, Tokyo, Japan; 5 Graduate School of Dental Science, Kyushu University, Fukuoka, Japan; 6 Monell Chemical Senses Center, Philadelphia, PA USA

**Keywords:** Gamma-aminobutyric acid, Taste buds, Glutamate decarboxylase, Taste mixture, Sour, Sweet

## Abstract

Taste buds contain 2 types of GABA-producing cells: sour-responsive Type III cells and glial-like Type I cells. The physiological role of GABA, released by Type III cells is not fully understood. Here, we investigated the role of GABA released from Type III cells using transgenic mice lacking the expression of GAD67 in taste bud cells (*Gad67*-cKO mice). Immunohistochemical experiments confirmed the absence of GAD67 in Type III cells of *Gad67*-cKO mice. Furthermore, no difference was observed in the expression and localization of cell type markers, ectonucleoside triphosphate diphosphohydrolase 2 (ENTPD2), gustducin, and carbonic anhydrase 4 (CA4) in taste buds between wild-type (WT) and *Gad67*-cKO mice. Short-term lick tests demonstrated that both WT and *Gad67*-cKO mice exhibited normal licking behaviors to each of the five basic tastants. Gustatory nerve recordings from the chorda tympani nerve demonstrated that both WT and *Gad67*-cKO mice similarly responded to five basic tastants when they were applied individually. However, gustatory nerve responses to sweet–sour mixtures were significantly smaller than the sum of responses to each tastant in WT mice but not in *Gad67*-cKO mice. In summary, elimination of GABA signalling by sour-responsive Type III taste cells eliminates the inhibitory cell–cell interactions seen with application of sour–sweet mixtures.

## Introduction

Taste bud cells, especially Type II and Type III cells, have a critical role in detecting chemical compounds in the oral cavity. Type II cells express taste receptors for sweet, bitter, and umami (and salty) taste, and they transmit this information to the gustatory nerve fibers via channel synapses [46]. The transmitter released from Type II cells has been identified as ATP [15, 22, 33]. On the other hand, Type III cells express the sour taste receptor, otopetrin 1 (OTOP1) [48, 62], and conventional synapses may be used for transmitting information from Type III cells to gustatory nerve fibers. One of the transmitters used in this synaptic transduction process may be serotonin, as serotonin is released from Type III cells in response to sour taste stimuli [21, 23], and serotonin activates gustatory nerve fibers via 5-HT3 receptors [30].

Type III taste cells release not only serotonin but also norepinephrine and γ-aminobutyric acid (GABA) in response to taste stimuli [20, 21]. Although taste-induced ATP release from Type III taste cells has not been detected in previous studies [22, 33, 38], ATP might also function as a neurotransmitter released from Type III cells to the gustatory nerve because purinergic transmission is necessary for taste responses to various tatstants including sour compounds [3, 5, 15]. Among them, GABA mainly acts as an inhibitory transmitter in the central nervous system. GABA is produced by decarboxylation of glutamate, mediated by glutamate decarboxylase (GAD) [14]. There are two isoforms of GAD, GAD65 (GAD2) and GAD67 (GAD1). In taste buds, GAD65 is mainly expressed in Type I cells [13, 29], which are thought to have a glial-like function. In contrast, GAD67 is selectively expressed in Type III taste cells [11, 49], most of which respond to sour tastants such as HCl, citric acid and acetic acid [19, 57]. The physiological role of GABA released from Type III cells is, however, still not elucidated.

As a neurotransmitter, GABA acts on two main classes of GABA receptors, GABA_A_ and GABA_B_ receptors [4]. GABA_A_ receptors are ionotropic receptors that allow the flux of Cl^−^ ions according to electrochemical gradients. In most neurons, this would be an inward Cl^−^ flux leading to hyperpolarization. But in immature neurons and some ganglion cells, intracellular Cl^−^ is high and so activation of GABA_A_ receptors may depolarize a cell [31, 51]. GABA_B_ receptors are metabotropic receptors that function to inhibit the excitability of neurons by opening K^+^ channels or through other pathways [17]. These GABA receptors are expressed in both taste bud cells [13] and gustatory afferent neurons of the geniculate ganglion [12]. Therefore, GABA released from Type III cells might affect both taste bud cells and gustatory nerve fibers. Of particular interest are GABA_A_ receptors on the gustatory afferent neurons, as GABA could function as an excitatory neurotransmitter if these neurons maintain a high level of Cl^−^ concentration similar to immature neurons [26, 53]. Thus, GABA could be a candidate neurotransmitter linking sour taste cells to corresponding gustatory afferent fibers. In addition, GABA could function as a local transmitter to exert paracrine interactions in taste buds [39], as a previous study demonstrated that forced activation of Type III cells using optogenetic techniques reduced signal output during gustatory stimulation [50].

In this study, we investigate the function of GABA in Type III cells using transgenic mice lacking the expression of GAD67 in taste bud cells. We employed immunohistochemistry, behavioral lick tests, and chorda tympani nerve recordings. Our findings suggest that GABA in Type III cells does not contribute to signal transmission from Type III cells to gustatory nerve fibers. However, there is a possibility that it functions as an inhibitory transmitter involved in cell–cell communication within taste buds.

## Materials and methods

### Animals

All experimental procedures were performed in accordance with the National Institutes of Health Guide for the Care and Use of Laboratory Animals and approved by the committee for Laboratory Animal Care and Use at Okayama University, Japan. The subjects were adult (> 8 weeks old) male and female wild-type (WT, C57BL/6 J), *Gad67*^GFP/+^ [B6.Cg-Gad1 < Tm1.1Tama >] [45], *Gad67*^flox/flox^ [B6.Cg-Gad1 < Tm2 >] [35], *Krt5*^Cre^*Gad67*^flox/flox^, *Krt5*^Cre^*Gad67*^flox/GFP^, *Krt5*^Cre^*Gad67*^flox/flox^*Trpv1*^−/−^, *Krt5*^Cre^*Gad67*^GFP/+^*Rosa26*^*lsl−Tom/lsl−Tom*^ mice. *Krt5*^Cre^*Gad67*^flox/flox^ mice were generated by crossing *Krt5*^Cre^ mice [B6.Cg-Tg(Krt5-Cre)1Tak] [47] with *Gad67*^flox/flox^ mice. *Krt5*^Cre^*Gad67*^flox/GFP^ mice were generated by crossing *Krt5*^Cre^*Gad67*^flox/flox^ mice with *Gad67*^GFP/+^ mice. *Krt5*^Cre^*Gad67*^flox/flox^*Trpv1*^−/−^ mice were generated by crossing *Krt5*^Cre^*Gad67*^flox/flox^ mice with *Trpv1*^−/−^ mice [B6.Cg-Trpv1 < tm1Jul >] [7]. *Krt5*^Cre^*Gad67*^GFP/+^*Rosa26*^*lsl−Tom/lsl−Tom*^ mice were generated by crossing *Krt5*^Cre^ mice, *Gad67*^GFP/+^ mice and *Rosa26*^lsl−Tom/lsl−Tom^ mice [32]. All strains of mice have a WT background with C57BL/6 J mice backcrossed for at least 5 generations. Mice were housed under a 12:12-h light–dark cycle (lights on 0800-2000 h) and had ad libitum access to tap water and food pellets (MF, Oriental yeast co., Tokyo, Japan).

### Histology & immunohistochemistry

The immunohistochemical procedures were modified from those reported previously [34, 58]. *Krt5*^Cre^*Gad67*^GFP/+^*Rosa26*^*lsl−Tom/lsl−Tom*^ mice (*n* = 3) were used in histological experiments. *Gad67*^GFP/+^ mice (*n* = 6) and *Krt5*^Cre^*Gad67*^flox/GFP^ mice (*n* = 6) were used as experimental subjects for immunohistochemistry. Animals were sacrificed by exposure to CO_2_. For immunohistochemical analysis of fungiform taste buds, the anterior tongue was removed and administrated with 100 μl of Tyrode solution containing 0.25 mg/ml elastase (Elastin Products, MO, USA) to peel the tongue epithelium. The peeled tongue epithelium bisected along the sagittal plane and each half was pinned out in a Sylgard-coated culture dish and was fixed in phosphate buffer saline with 4% paraformaldehyde (PFA/PBS). For analysis of circumvallate taste buds and histological analysis of fungiform taste buds, dissected posterior or anterior part of tongues were fixed in 4% PFA/PBS for 45 min at 4 °C. After dehydration in sucrose solutions (15% for 1 h and 30% for 2 h at 4 °C), frozen blocks of fixed tongues were embedded in OCT compound (Sakura Finetek, Tokyo, Japan) and cut into 10 µm-thick sections, which were mounted on silane-coated glass slides. Both fungiform and circumvallate sections of *Krt5*^Cre^*Gad67*^GFP/+^*Rosa26*^*lsl−Tom/lsl−Tom*^ mice were directory observed using a laser scanning microscope (LSM780, Carl Zeiss, Oberkochen, Germany or FV-300, Olympus, Tokyo, Japan) after washing with tris-buffered saline (TBS). Images were then analyzed with Zen software (Carl Zeiss) or FLUOVIEW software (Olympus).

For immunostaining, both fungiform and circumvallate samples were washed with TBS, treated with Blocking One-P (Nacalai tesque, Kyoto, Japan) for 1 h at room temperature, and then incubated overnight at 4 °C with primary antibodies against GAD67 (goat IgG, 1:100, AF2086, R&D systems, Minneapolis, MN, USA; RRID = AB_2107724), ENTPD2 (sheep IgG, 1:400, AF5797, R&D systems; RRID = AB_10572702), gustducin (goat-IgG, 1:200, Aviva systems Biology, San Diego, CA, USA; RRID = AB_10882823) or CA4 (goat-IgG, 1:400, AF2414, R&D systems; RRID = AB_2070332). After washing with TBS, samples were incubated with secondary antibodies against goat IgG (Alexa Fluor 568 donkey anti-goat IgG H + L, 1:200, Thermo Fisher Scientific, Waltham, MA, USA; RRID = AB_2534104) or Sheep IgG (donkey anti sheep IgG H&L Alexa Fluor 568, 1:200, Abcam, Cambridge, UK; RRID = AB_2892984). The GFP fluorescent and fluorescent-labeled taste cells were observed with a laser scanning microscope (LSM780 or FV-300) and analyzed with Zen software (Carl Zeiss) or FLUOVIEW software (Olympus).

### Solutions

Tyrode solution contained: NaCl, 140 mM; KCl, 5 mM; CaCl_2_, 1 mM; MgCl_2_, 1 mM; NaHCO_3_, 5 mM; HEPES, 10 mM; Glucose, 10 mM; sodium pyruvate, 10 mM; pH adjusted to 7.4 with NaOH. Taste solutions were as follows: 100 mM NH_4_Cl, 10–1000 mM sucrose (Suc), 10–1000 mM NaCl, 0.3–100 mM citric acid, 0.3–100 mM HCl, 0.3–100 mM acetic acid, 10–300 mM monopotassium glutamate (MPG), 10–300 mM monosodium glutamate (MSG), 0.01–20 mM quinine-HCl (QHCl), 10 µM capsaicin. Sweet–sour mixtures (500, 1000 mM glucose, 500 mM sucrose, 10, 20 mM sucralose or 10 mM saccharin + 10 mM HCl) were also used in gustatory nerve recordings. Chemicals were purchased from FUJIFILM Wako Pure Chemical Corporation (Osaka, Japan), Nakarai tesque (Kyoto, Japan) or Sigma-Aldrich (St. Louis, MO, USA).

### Gustatory nerve recording

Whole nerve responses to lingual application of tastants were recorded from the chorda tympani (CT) nerve following previously described methods [28, 59]. WT (*n* = 20), *Krt5*^Cre^*Gad67*^flox/flox^ (*n* = 4) and *Krt5*^Cre^*Gad67*^flox/GFP^ mice (*n* = 13) were used as experimental subjects. Mice were anesthetized by an injection of a combination anesthetic (0.3 mg/kg of medetomidine, 4.0 mg/kg of midazolam, and 5.0 mg/kg of butorphanol) and maintained at a surgical level of anesthesia with supplemental injections of the same combination (0.15 mg/kg of medetomidine, 2.0 mg/kg of midazolam, and 2.5 mg/kg of butorphanol approximately every 2 h). The anesthetic level was evaluated by testing the withdrawal reflex to a paw pinch. Under anesthesia, the trachea of each mouse was cannulated and then the mouse was fixed in the supine position with a head holder to allow dissection of the CT nerve. The right CT nerve was dissected free from surrounding tissues after removal of the pterygoid muscle and cut at the point of its entry into the bulla. The entire nerve was placed on an Ag/AgCl electrode with an indifferent electrode placed in nearby tissue. Neural activities were amplified using the DAM80 amplifier (World Precision Instruments, Sarasota, FL, USA) and monitored on an oscilloscope. Whole nerve responses were integrated with a time constant of 1.0 s and recorded on a computer using a PowerLab system (PowerLab/sp4; AD Instrument, Bella Vista, Australia). The anterior one-half of the tongue was enclosed in a flow chamber of silicone rubber. Taste solutions were delivered to the tongue by gravity flow for 30 s. The tongue was washed with distilled water (DW) for an interval of approximately 1 min between successive stimulation. Only responses from stable recordings were used for data analysis. At the end of experiments, animals were euthanized by administration of an overdose of the anesthetic.

### Short term lick response

Behavioral lick responses to various tastants were recorded as described previously [54]. WT (*n* = 7), *Gad67*^flox/flox^ (*n* = 11), *Krt5*^Cre^*Gad67*^flox/flox^ (*n* = 9), and *Krt5*^Cre^*Gad67*^flox/flox^*Trpv1*^−/−^ mice (*n* = 7), housed in individual cages, were used as experimental subjects. On day 1 of training, each animal was water-deprived for 12 h and then placed in the test cage and given free access to distilled water (DW) during the 1 h session. Days 2–5 comprised training sessions, during which animals were trained to drink DW on an interval schedule consisting of 5-s periods of DW presentation alternating with 10-s intertrial intervals. From day 6, the numbers of licks for each taste solution and DW were counted during the first 5 s after the animal’s first lick, using a lick meter (Yutaka Electronics Co., Gifu, Japan). The test solutions used were 30–1000 mM NaCl, 30–1000 mM sucrose, 0.01–1 mM quinine-HCl, 10–300 mM sodium glutamate, 1–100 mM citric acid, and 1–100 mM HCl. One tastant, at varying concentrations, was tested on any given test day. To examine lick responses to preferred solutions (sucrose and MSG), mice were deprived of both food and water 12 h before the experiment. On each test day, mice were given test solutions with concentrations of descending order (from highest concentration to DW) in the first trial then randomized order in subsequent trials. To examine lick responses to aversive solutions (NaCl, QHCl, citric acid and HCl), mice were deprived of water 12 h before the beginning of experiment. On each test day, mice were given test solutions in ascending concentration order (from DW to highest concentration) in the first trial and then randomized order for subsequent trials. The number of lick trials for each solution was at least three, and their values were averaged for data analysis.

### Data analysis

For immunohistochemical data, differences between genotypes were statistically analyzed using Fisher’s exact test. In the analysis of whole nerve responses, integrated whole nerve response magnitudes were measured at 5–25 s after stimulus onset, averaged and normalized to responses to 100 mM NH_4_Cl to account for mouse-to-mouse variations in absolute responses. This relative response was used for statistical analysis. For nerve recordings, differences among concentrations of each tastant and differences among genotypes were statistically analyzed by using two-way ANOVA. For short term lick responses to HCl, citric acid, NaCl, quinine-HCl, sucrose and MSG, differences among concentrations of each tastant and differences among genotypes were statistically analyzed by repeated measures two-way ANOVA. For short term lick responses to 10 µM capsaicin, difference among genotypes was statistically analyzed by one-way ANOVA and post hoc Tukey highest-significant-difference (HSD) test. For responses to the sweet–sour mixture, differences between genotypes or between the sum of responses and responses to the mixture were statistically analyzed by Student’s t-test. All statistical analyses were performed using Jamovi software (ver. 2.3.21, https://www.jamovi.org/). *P*-values < 0.05 were considered significant.

## Results

### Expression of GAD67 in taste tissues

**Fig. 1:**
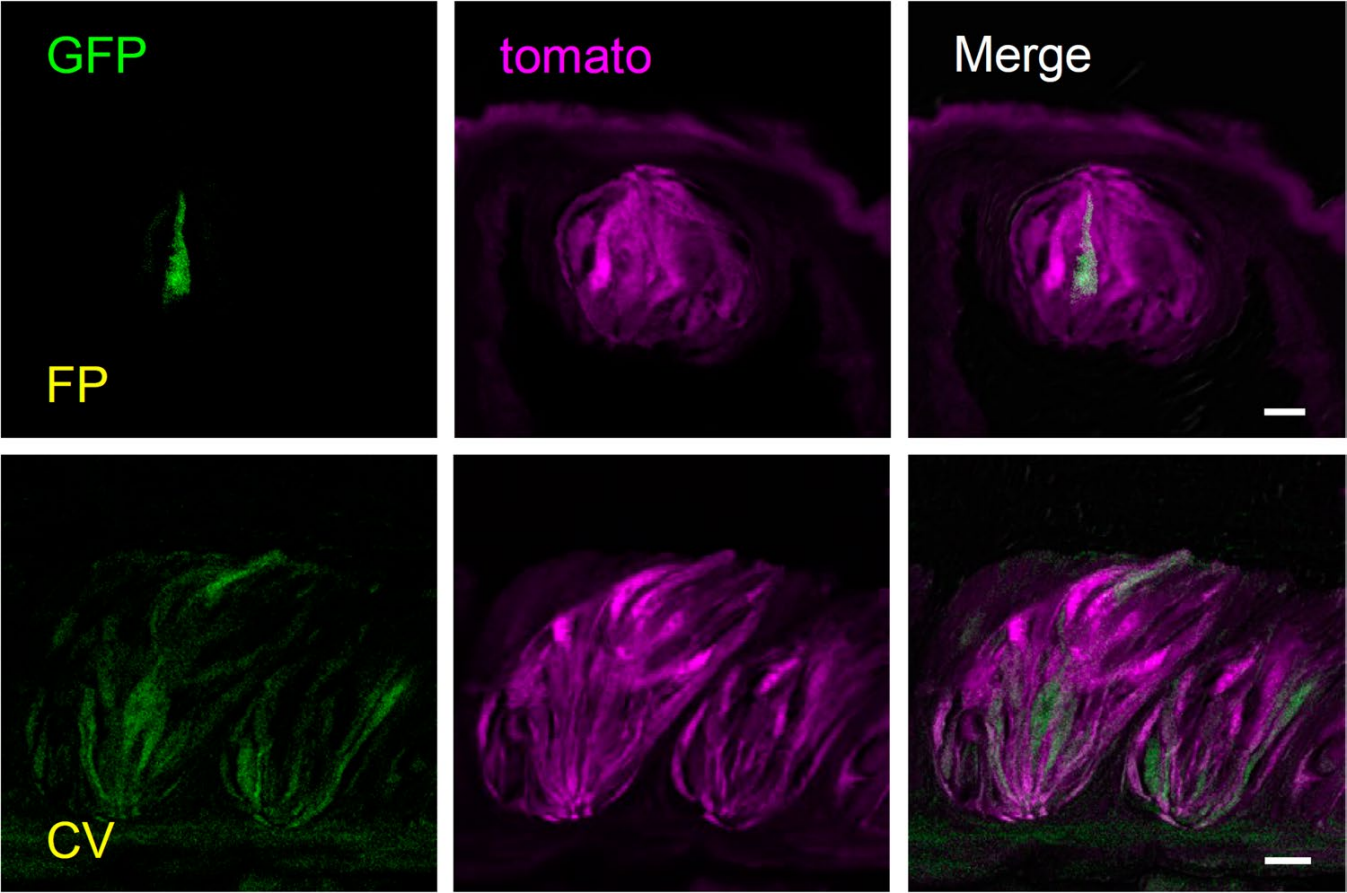
*Krt5*^Cre^ mice were useful to induce gene recombination in taste tissues. Detection of fluorescent proteins in taste tissues of *Krt5*^Cre^*Gad67*^GFP/+^*Rosa26*^lsl−Tom/lsl−Tom^ mice. Green: GFP fluorescence. Magenta: Tomato fluorescence. CV: circumvallate papillae. Scale bar, 10 µm

A previous study demonstrated that basal keratinocytes expressing Krt5 and Krt14 were identified as taste progenitor cells [37]. In addition, *Krt5*^CreERT2^ mice were used to induce gene recombination in taste tissue [36]. Therefore, we considered that *Krt5*^cre^ mice expressing Cre recombinase in Krt5-expressing cells could be used to generate *Gad67*-cKO mice (*Krt5*^Cre^*Gad67*^flox/flox^) by crossing these mice with *Gad67*^flox/flox^ mice. To test whether *Krt5*^cre^ mice could be used to generate a cKO model, we first examined reporter gene expression in taste tissues of *Krt5*^Cre^*Gad67*^GFP/+^*Rosa26*^*lsl−Tom/lsl−Tom*^ mice (Fig. 1). In both fungiform and circumvallate papillae, almost all taste bud cells and surrounding tissues were labeled with the fluorescent protein tdTomato and some taste bud cells were marked with GFP, indicating *Gad67*-expressing taste cells.

GAD67 is expressed in Type III taste cells in mice [11, 49] and GFP-positive taste cells of *Gad67*^GFP^ mice respond to sour taste stimuli [57]. In addition, GABA is released from type III taste cells in response to sour taste stimuli [21]. Therefore, GAD67 and GABA may play roles in Type III taste cells (sour-sensitive taste cells). To investigate the functions of GAD67 and GABA in Type III taste cells, we produced conditional *Gad67*-KO mice (*Gad67*-cKO) lacking the expression of GAD67 in Type III taste cells, because conventional *Gad67*-KO mice were lethal at birth [1]. By crossing *Krt5*^cre^ mice and *Gad67*^flox/flox^ mice, we generated *Gad67*-cKO mice (*Krt5*^Cre^*Gad67*^flox/flox^). Since a cDNA-encoding EGFP was targeted to the locus encoding GAD67 with disruption of the coding sequence for GAD67 in *Gad67*^GFP^ mice [45], we also generate triple mutant mice (*Krt5*^Cre^*Gad67*^flox/GFP^) and used these mice also as *Gad67*-cKO mice.

**Fig. 2:**
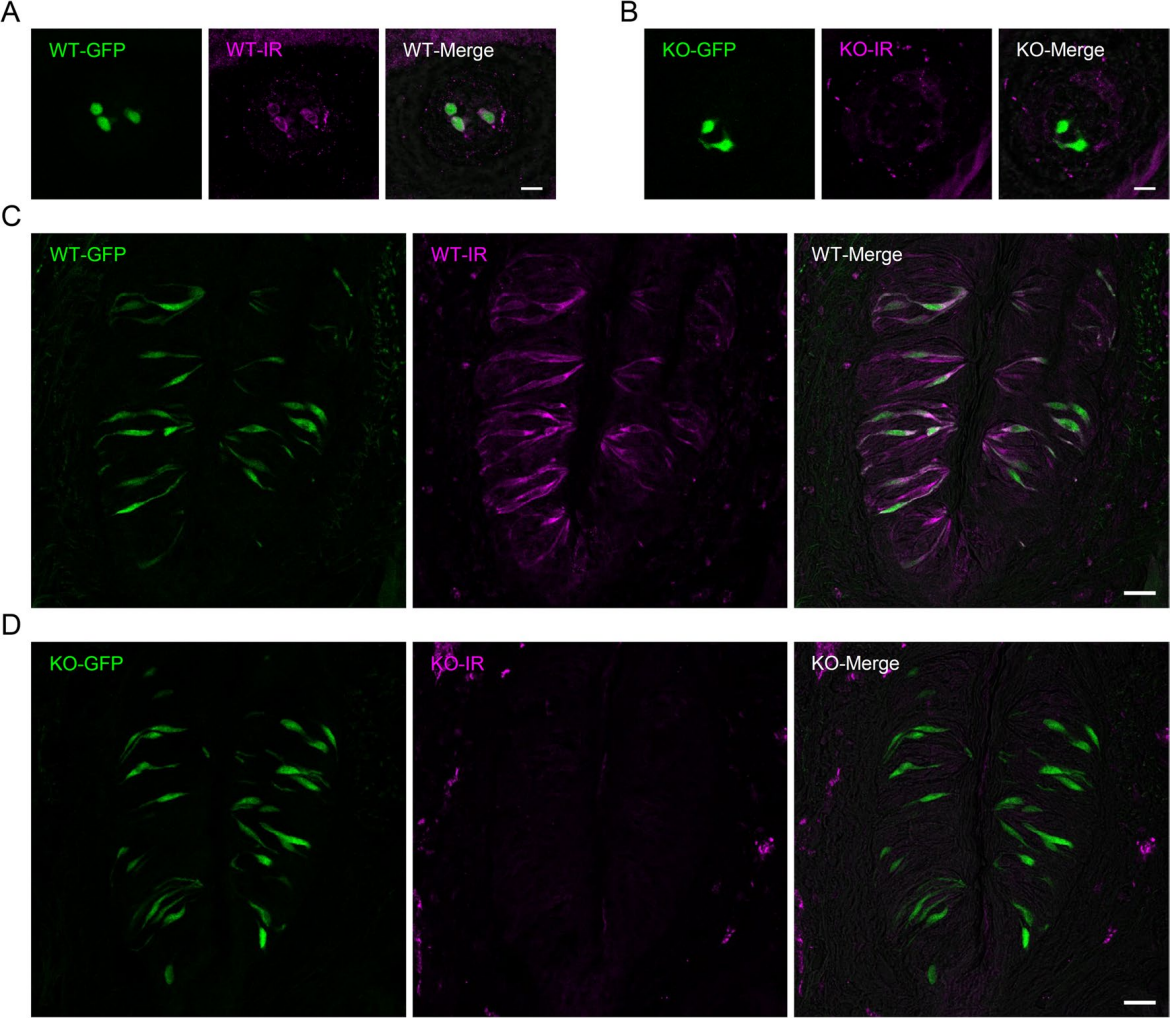
Lack of expression of GAD67 protein in taste buds of *Gad67*-cKO mice. Immunohistochemical detection of GAD67 in taste buds of *Gad67*^GFP/+^ mice (WT) and *Krt5*^Cre^*Gad67*^GFP/flox^ mice (KO). **A**-**D**. Immunostaining for GAD67 and GFP expression in fungiform (**A**, **B**) and circumvallate papillae (**C**, **D**) of a *Gad67*^GFP/+^ mouse (**A**, **C**) and a *Krt5*^Cre^*Gad67*.^GFP/flox^ mouse (**B**, **D**). Summarized data are shown in Table 1. Green: GFP fluorescence, Magenta: immunoreactivity (IR) for GAD67. *N* = 3 animals. Scale bar, 10 µm (**A**, **B**) or 20 µm (**C**, **D**)

The expression of GAD67 was examined by immunohistochemistry. We used *Gad67*^GFP/+^ mice as WT controls and *Krt5*^Cre^*Gad67*^flox/GFP^ mice as *Gad67*-cKO. In these mice, GFP positive taste cells were identified as Type III taste cells. GAD67 immunoreactivity was observed in GFP-positive taste cells of fungiform papillae (85.5%) and circumvallate papillae (94.5%) of *Gad67*^GFP/+^ mice (Fig. 2A and C) although some GAD67 immunoreactive cells did not express GFP. In contrast, GAD67 immunoreactivity was almost completely lost in *Krt5*^Cre^*Gad67*^flox/GFP^ mice (Fig. 2B and D). The expression of GAD67 was significantly different between WT and *Gad67*-cKO mice (Table 1).

**Table 1:** Summary of immunohistochemical data

	FP			CV		
	*Gad67*^GFP/+^	*Krt5*^Cre^*Gad67*^GFP/flox^	Fisher’s exact test	*Gad67*^GFP/+^	*Krt5*^Cre^*Gad67*^GFP/flox^	Fisher’s exact test
GAD67-GFP	83	89		469	425	
GAD67-IR	75	1		517	4	
Merge	71	1	*P* < 0.001	443	0	*P* < 0.001
GAD67-GFP	74	71		451	475	
GNAT3-IR	232	206		677	702	
Merge	0	0	*P* = 0.702	0	0	*P* = 0.865
GAD67-GFP	79	74		462	449	
CA4-IR	83	82		543	503	
Merge	79	74	*P* = 0.966	419	411	*P* = 0.797

Each data from 3 animals

**Fig. 3:**
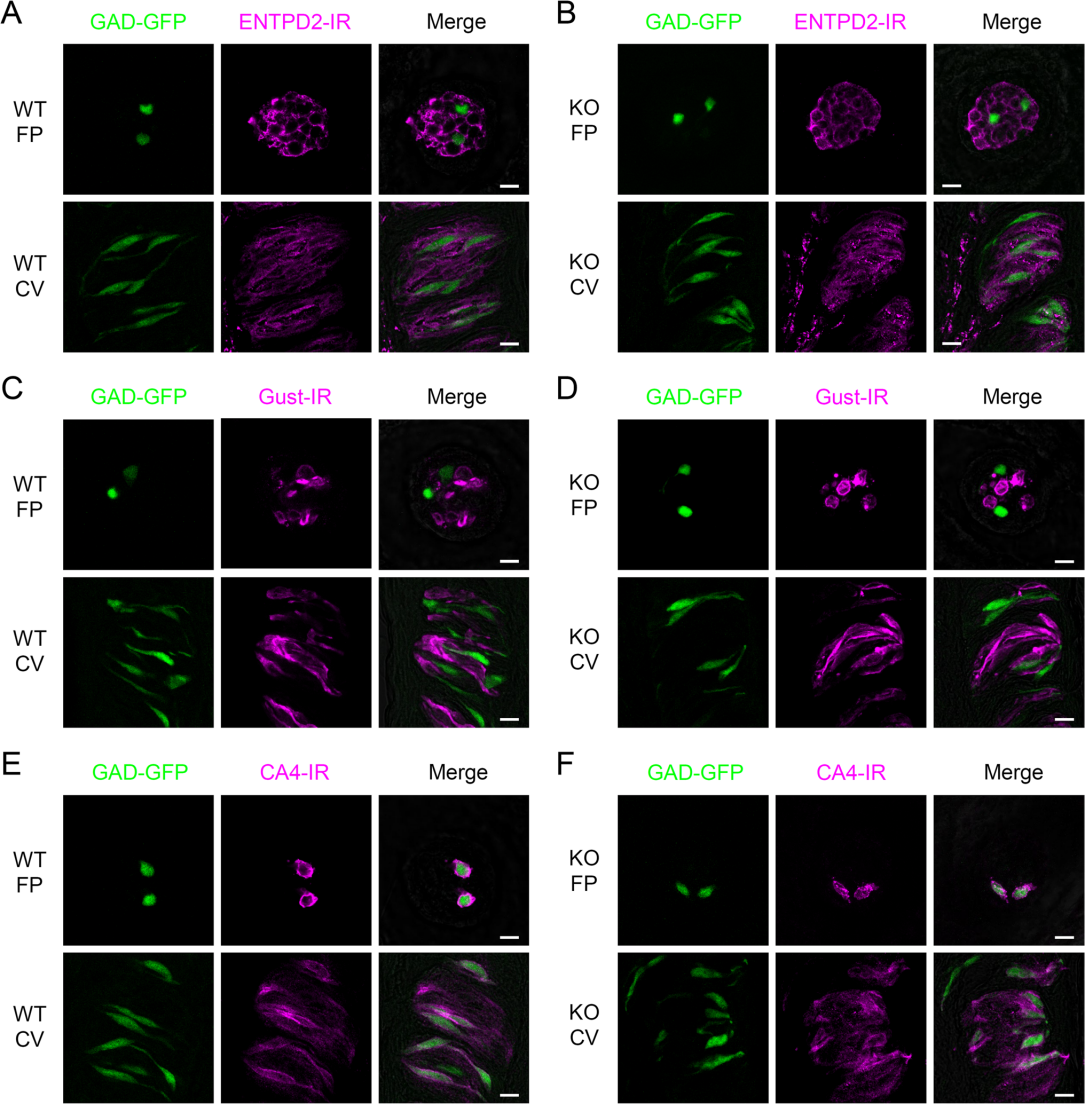
Expression of taste cell markers was not impaired in taste buds of *Gad67*-cKO mice. Immunohistochemical detection of taste cell markers in taste buds of *Gad67*^GFP/+^ mice (WT) and *Krt5*^Cre^*Gad67*^GFP/flox^ mice (KO). Immunostaining for ENTPD2 and GFP expression in fungiform (FP) and circumvallate papillae (CV) of a *Gad67*^GFP/+^ mouse (**A**) and a *Krt5*^Cre^*Gad67*^GFP/flox^ mouse (**B**). Immunostaining for Gustducin and GFP expression in FP and CV of a *Gad67*^GFP/+^ mouse (**C**) and a *Krt5*^Cre^*Gad67*^GFP/flox^ mouse (**D**). Immunostaining for CA4 and GFP expression in FP and CV of a *Gad67*^GFP/+^ mouse (**E**) and a *Krt5*^Cre^*Gad67*^GFP/flox^ mouse (**F**). Summarized data are shown in Table 1. Green: GFP fluorescence, Magenta: immunoreactivity (IR) for ENTPD2, Gustducin or CA4. *N* = 3 animals. Scale bar, 10 µm

We also examined the expression of other taste cell markers: ENTPD2 as a Type I cell marker [2] (Fig. 3A, B), Gustducin as a Type II cell marker [55] (Fig. 3C, D) and CA4 as a Type III cell marker [8] (Fig. 3E, F) in *Gad67*^GFP/+^ mice and *Krt5*^Cre^*Gad67*^flox/GFP^ mice. Expression of these cell type markers was observed in both *Gad67*^GFP/+^ mice and *Krt5*^Cre^*Gad67*^flox/GFP^ mice. Expression of gustducin and CA4 was not significantly different between WT and *Gad67*-cKO mice (Table 1). These results suggest that *Gad67*-cKO mice lacked the expression of GAD67 in taste tissue. In addition, the lack of GAD67 expression in taste tissue may not affect the expression of other taste cell markers, such as ENTPD2, Gustducin and CA4.

### Gustatory nerve responses to single tastant

**Fig. 4:**
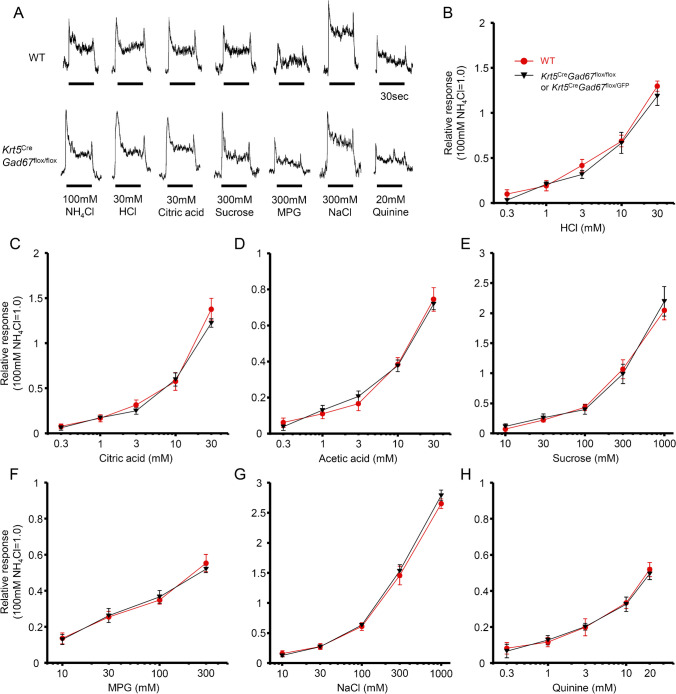
Lack of GAD67 in taste buds did not affect gustatory nerve responses to single tastants. **A**. Sample recordings of chorda tympani nerve responses of WT (upper) and *Gad67*-cKO mouse (lower). Taste stimuli were NH_4_Cl (100 mM), HCl (30 mM), citric acid (30 mM), sucrose (300 mM), MSG (300 mM), NaCl (300 mM), quinine (20 mM). **B**-**H**. Concentration–response relationships of chorda tympani nerve responses of WT mice (red circle) and *Gad67*-cKO mice (black triangle) for HCl (WT: *n* = 9, cKO: *n* = 10), citric acid (WT: *n* = 11, cKO: *n* = 9), acetic acid (WT: *n* = 8, cKO: *n* = 9), sucrose (WT: *n* = 10, cKO: *n* = 9), MPG (WT: *n* = 9, cKO: *n* = 9), NaCl (WT: *n* = 12, cKO: *n* = 11), quinine (WT: *n* = 8, cKO: *n* = 9). Gustatory nerve responses were normalized to the response to 100 mM NH_4_Cl. Values indicated are means ± S.E.M. Statistical differences were analyzed by two-way ANOVA tests (Table 2)

Next, we examined whether the lack of GAD67 in taste tissues affects gustatory nerve responses to basic taste stimuli (Fig. 4). If gustatory nerve terminals were activated by GABA, then elimination of GAD67 from Type III cells should result in decreased responses to acid. We used C57BL/6 J mice as WT controls and *Krt5*^Cre^*Gad67*^flox/flox^ or *Krt5*^Cre^*Gad67*^flox/GFP^ mice as *Gad67*-cKO. We recorded CT nerve responses to sour (HCl, citric acid, and acetic acid), sweet (sucrose), umami (MPG), salty (NaCl) and bitter (quinine) tastants. Since GAD67 is expressed in sour-sensitive Type III taste cells, we first focused on gustatory nerve responses to sour tastants (Fig. 4, A-D). However, we did not observe any significant difference in CT nerve responses to sour tastants between WT and *Gad67*-cKO mice (Table 2). In addition, CT nerve responses of *Gad67*-cKO mice to other tastants (sucrose, MPG, NaCl, and quinine) were almost similar to those of WT mice (Fig. 4E–H). These results suggest that the lack of GAD67 in taste tissue does not affect gustatory nerve responses to each of five basic tastes. It is notable that a previous study demonstrated that Type III cells contribute to responses to NH_4_Cl [31]. However, CT nerve responses to NH_4_Cl were not significantly different between WT and *Gad67*-cKO mice when NH_4_Cl responses were normalized to CT nerve responses to 300 mM sucrose [WT: 1.11 ± 0.18 (*n* = 8), cKO: 1.22 ± 0.18 (*n* = 9), *P* > 0.1, Student’s t-test].

**Table 2:** Two-way ANOVA results for CT nerve recordings

Tastant	Effect	Degree of Freedom	F Value	*p* Value
HCl	genotype	1.82	1.78	0.186
	concentration	4.82	91.4	< 0.001***
	interaction	4.82	0.315	0.867
Citric acid	genotype	1.89	2.41	0.124
	concentration	4.89	117.8	< 0.001***
	interaction	4.89	0.803	0.527
Acetic acid	genotype	1.74	0.001	0.972
	concentration	4.74	120.8	< 0.001***
	interaction	4.74	0.328	0.858
Sucrose	genotype	1.79	0.109	0.743
	concentration	4.79	96.3	< 0.001***
	interaction	4.79	0.25	0.909
MPG	genotype	1.64	0.021	0.886
	concentration	3.64	53.6	< 0.001***
	interaction	3.64	0.219	0.883
NaCl	genotype	1.102	0.646	0.423
	concentration	4.102	387	< 0.001***
	interaction	4.102	0.389	0.816
quinine	genotype	1.75	0.099	0.754
	concentration	4.75	53.2	< 0.001***
	interaction	4.75	0.084	0.987

**: *P* < 0.001

### Behavioral lick responses to single tastants

**Fig. 5:**
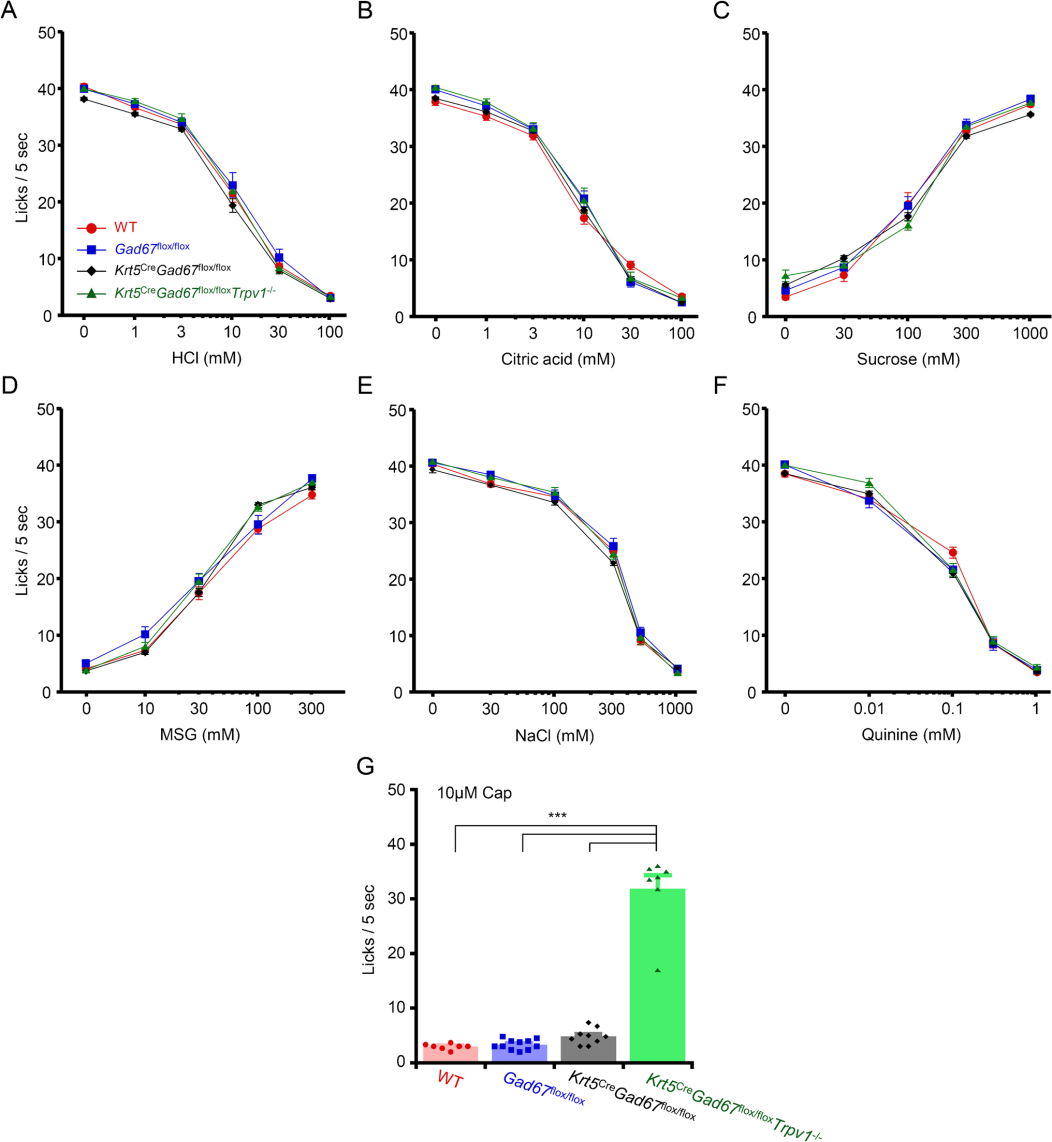
Lack of GAD67 in taste buds did not affect behavioral lick responses to single tastants. Number of licks of 30–1000 mM NaCl (**A**), 0.01–3 mM quinine (**B**), 30–1000 mM sucrose (**C**), 10–300 mM MSG (**D**), 1–100 mM HCl (**E**), 1–100 mM citric acid (**F**) and 10 µM capsaicin in the short-term (5 s) lick test. Red circle: WT mice (*n* = 7), Blue rectangle: *Gad67*^flox/flox^ mice (*n* = 11), Green diamond: *Krt5*^Cre^*Gad67*^flox/flox^ mice (*n* = 9), Black triangle: *Krt5*^Cre^*Gad67*^flox/flox^*Trpv1*.^−/−^ mice (*n* = 7). Values indicated are means ± S.E.M. Statistical differences were analyzed by two-way ANOVA tests (Table 3) or one-way ANOVA with post hoc Tukey HSD test. ***: *P* < 0.001

We next performed short term (5 s) lick tests to examine whether our *Gad67*-cKO mice have any behavioral impairment in taste behavior to the five basic tastes. A previous study demonstrated that aversive responses to sour (oral acid) were mediated by both taste and somatosensory neural pathways, because *Otop1*-KO mice with bilateral injection of resiniferatoxin (RTX) in the trigeminal ganglia showed decreased aversive responses to sour stimuli, although *Otop1*-KO mice and RTX treated mice avoided sour stimuli similarly to WT mice [62]. Therefore, in this study, we used *Krt5*^Cre^*Gad67*^flox/flox^*Trpv1*^−/−^ mice in addition to WT, *Gad67*^flox/flox^, *Krt5*^Cre^*Gad67*^flox/flox^ mice to identify any deficiency in sour taste responses of *Gad67*-cKO mice (Fig. 5). In line with our results from CT nerve recordings, short term lick responses to sour tastants (HCl and citric acid) were not significantly different among WT, *Gad67*^flox/flox^, *Krt5*^Cre^*Gad67*^flox/flox^, and* Krt5*^Cre^*Gad67*^flox/flox^*Trpv1*^−/−^ mice (Fig. 5A, B, Table 3). In addition, short term lick responses to other tastants (sucrose, MSG, NaCl, and QHCL) were almost similar among WT, *Gad67*^flox/flox^, *Krt5*^Cre^*Gad67*^flox/flox^, and* Krt5*^Cre^*Gad67*^flox/flox^*Trpv1*^−/−^ mice (Fig. 5C-F, Table 3). *Krt5*^Cre^*Gad67*^flox/flox^*Trpv1*^−/−^ mice showed a significant reduction in avoidance to capsaicin compared to WT, *Gad67*^flox/flox^, *Krt5*^Cre^*Gad67*^flox/flox^ mice (Fig. 5G) because *Krt5*^Cre^*Gad67*^flox/flox^*Trpv1*^−/−^ mice lacked the expression of TRPV1. Taken together, deletion of GAD67 in Type III taste cells did not lead to any taste deficiency in short term lick tests.

**Table 3:** Repeated two-way ANOVA results for short term lick tests

Tastant	Effect	Degree of Freedom	F Value	*p* Value
HCl	genotype	3.30	1.80	0.169
	concentration	5.150	1562	< 0.001***
	interaction	15.150	0.785	0.693
Citric acid	genotype	3.30	1.19	0.330
	concentration	5.150	1591	< 0.001***
	interaction	15.150	1.78	0.043*
NaCl	genotype	3.30	2.17	0.972
	concentration	5.150	1987	< 0.001***
	interaction	15.150	0.871	0.598
quinine	genotype	3.30	0.722	0.547
	concentration	4.120	2016	< 0.001***
	interaction	12.120	2.3	0.011*
Sucrose	genotype	3.30	0.532	0.664
	concentration	4.120	1418	< 0.001***
	interaction	12.120	3.56	< 0.001***
MSG	genotype	3.30	1.98	0.138
	concentration	4.120	1116	< 0.001***
	interaction	12.120	2.45	0.007**

*: *P* < 0.05, **: *P* < 0.01, ***: *P* < 0.001

### Gustatory nerve responses to sour–sweet mixture

**Fig. 6:**
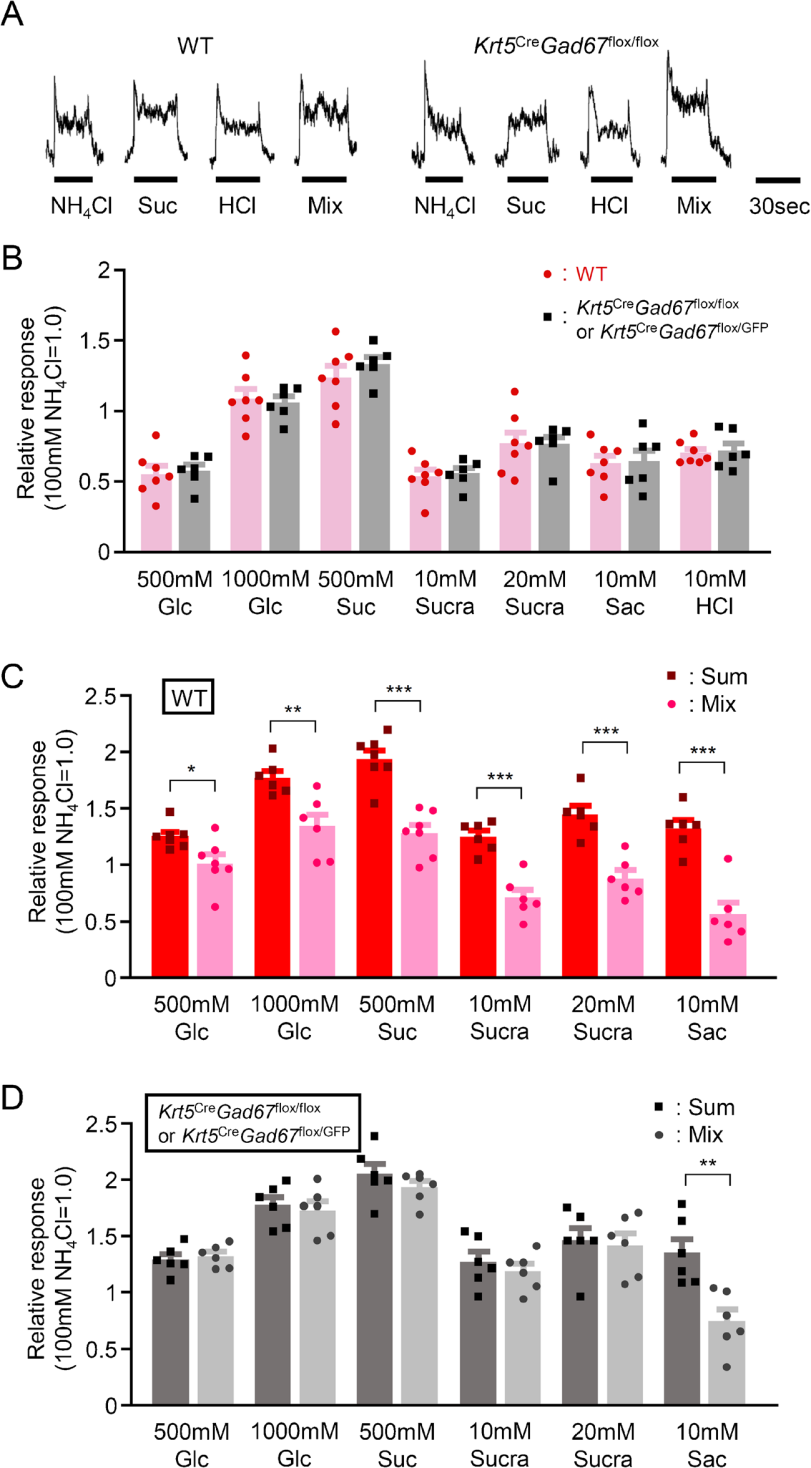
*Gad67*-cKO mice showed greater responses to sweet–sour mixtures. **A**. Sample recordings of chorda tympani nerve responses of WT (left) and *Gad67*-cKO mice (right). Taste stimuli were NH_4_Cl (100 mM), sucrose (Suc; 500 mM), HCl (10 mM), sucrose + HCl (Mix; 500 mM, 10 mM, respectively). **B**. Chorda tympani nerve responses to glucose (Glc; 500, 1000 mM), sucrose (Suc; 500 mM), Sucralose (Sucra, 10, 20 mM), saccharin (Sac; 10 mM) and HCl (10 mM) in WT (red) and *Gad67*-cKO mice (black). **C**, **D**. Comparison between chorda tympani nerve responses to sweet–sour mixtures (500, 1000 mM glucose, 500 mM sucrose, 10, 20 mM sucralose or 10 mM saccharin + 10 mM HCl, left, light color) and sum of sweet and sour responses (right, dark color) in WT (C) and *Gad67*-cKO mice (D). Gustatory nerve responses were normalized to the response to 100 mM NH_4_Cl. Values indicated are means ± S.E.M. Statistical differences were analyzed by Student’s t-test. *: *P* < 0.05, **: *P* < 0.01, ***: *P* < 0.001

GABA receptors are expressed in taste bud cells [13] and patch-clamp recordings from acutely dissociated rat taste cells demonstrated that GABA_A_ and GABA_B_ agonist elicited linear chloride and inwardly rectifying potassium currents, respectively, indicating that the taste cells can respond to GABA [6]. Moreover, the existence of negative cross-talk between GABA_A_ and P2X receptors has been documented in cultured rat dorsal root ganglion neurons [43]. Thus, GABA could exert paracrine actions in taste buds [39]. We tested this hypothesis by using sweet–sour mixtures in gustatory nerve recordings (Fig. 6). If GABA functions as an inhibitory transmitter from Type III cells to sweet-sensitive taste cells, the response to the mixture would be smaller than the sum of each individual response. We applied various sweeteners (glucose, sucrose, sucralose, saccharin) and a sour tastant (HCl), and then a mixture of them, recording CT nerve responses. Similar to CT nerve responses to other tastants, responses to single tastants were almost similar between WT and *Gad67*-cKO mice (Fig. 6A, B). Responses to mixtures (e.g. 500 mM sucrose + 10 mM HCl) were significantly smaller than sum of responses (e.g. response to 500 mM sucrose + response to 10 mM HCl) in WT mice (Fig. 6A, C), suggesting that there are inhibitory interactions between sweet and sour tastes in WT mice. In contrast, mixture responses in *Gad67*-cKO mice were not significantly different to sum of responses, except in the case of saccharin (Fig. 6A, D). Thus, sweet–sour interaction was likely lost in *Gad67*-cKO mice. One possible explanation for this exception could be that the sweet–sour interaction is mediated at the receptor level (TAS1R2 or TAS1R3). Acidity might affect the receptor-ligand binding between saccharin and TAS1R2 or TAS1R3, similar to the binding of miraculin and sweet receptors.

## Discussion

In this study, we investigated the role of GAD67 in Type III taste cells. It was reported that conventional *Gad67*-KO mice were lethal at birth [1]. On the other hand, our cKO model (*Krt5*^Cre^*Gad67*^flox/flox^ and *Krt5*^Cre^*Gad67*^GFP/flox^) appeared to develop normally; there was no observable abnormalities in growth and daily life behaviors. At the taste tissue level, the expression of GAD67 was abolished in both fungiform and circumvallate papillae in *Gad67*-cKO mice (Fig. 2). GABA is a major inhibitory neurotransmitter, but it also contributes to proliferation, migration, and dendritic maturation of neurons in the central nervous system [41, 42]. Thus, there is a possibility that GABA released from Type III cells contributes to development or morphology of taste bud cells. However, the expression of other cell type markers such as ENTPD2 (Type I) and gustducin (Type II) and as well as the Type III taste cell marker CA4 and GAD67-GFP, was not significantly different between WT and cKO mice (Fig. 3), suggesting that the lack of GAD67 in taste buds do not affect histological aspects of taste buds.

GABA_A_ receptors are expressed in gustatory afferent neurons of the geniculate ganglion [12]. Therefore, GABA released from Type III cells may affect the activity of gustatory afferent neurons. Since GABA_A_ receptors are Cl^−^ channel, the effect of GABA on neural activity depends on the intracellular Cl^−^ concentration of GABA_A_-expressing neurons. If the neuron maintains high intracellular Cl^−^ concentration, GABA could act as an excitatory neurotransmitter. In general, the intracellular Cl^−^ concentration of mature neurons is maintained low, therefore, GABA functions as an inhibitory transmitter. In any case, we would expect to observe some differences in gustatory nerve responses (especially to sour tastants) between WT and *Gad67*-cKO mice, if GABA functions as a neurotransmitter between Type III cell and gustatory nerve fibers. We demonstrated that CT nerve responses to sweet (sucrose), salty (NaCl), bitter (quinine), umami (MSG) and sour (HCl and citric acid) tastants were not significantly different between WT and *Gad67*-cKO mice (Fig. 4). These results indicate that GABA dose not play a substantial role in transmission of the signal from Type III (sour-sensitive) taste cells to corresponding gustatory nerve fibers. GABA could serve as a general trophic factor for afferent nerves to establish a connection between taste bud cells and afferent nerve fibers [9]. In our study, the lack of GAD67 in taste buds did not lead to any impairment in taste sensitivity to single modalities of basic tastes (Fig. 4). Thus, GABA produced by GAD67 in taste bud cells is not likely to function as a general trophic factor for the innervation of afferent nerve fibers.

In this study, we also examined short term lick responses to single modalities of taste stimuli. One of the main targets in this study was sour taste. Previous study demonstrated that aversive responses to sour stimuli were mediated by both taste and somatosensory neural pathway [62]. This was further confirmed in our recent study using mice with impairment of Type III taste cells and lacking TRPV1[56], although *Trpv1*-KO mice did not show any significant difference in behavioral responses compared to WT mice to sour stimuli [40, 60]. Therefore, we generated double KO mice lacking the expression of GAD67 in taste tissues and TRPV1 in the whole body (*Krt5*^Cre^*Gad67*^flox/flox^*Trpv1*^−/−^ mice) for behavioral tests. However, we found that short term lick responses to sweet, salty, bitter, umami or sour tastants were almost similar among WT, *Gad67*^flox/flox^, *Krt5*^Cre^*Gad67*^flox/flox^ and *Krt5*^Cre^*Gad67*^flox/flox^*Trpv1*^−/−^ mice (Fig. 5). Together with our data from chorda tympani nerve responses to sweet, salty, bitter, umami or sour tastans, GABA in Type III cells might have no significant role in signal transmission from taste cells to gustatory nerve fibers.

As we demonstrated, GABA in Type III cells may not have a direct effect on gustatory nerve fibers and the perception of sour tastants. In addition, GAD67 in taste buds is not likely to contribute to development and morphology of other types of taste bud cells. What is the function of GABA released from Type III cells? A previous study demonstrated that GABA may function as an inhibitory transmitter within taste buds [13]. In this case, deletion of *Gad67* in taste buds may not affect taste sensitivity to single modalities of taste stimulus. However, responses to taste mixtures such as sweet and sour tastes might be different between WT and *Gad67*-cKO mice. Indeed, our results demonstrated that response to sweet–sour mixture was smaller than the sum of these responses in WT mice but not in *Gad67*-cKO mice except in the case of saccharin (Fig. 6). Therefore, *Gad67*-cKO mice might lose the peripheral inhibition of responses to sweet tastants by sour taste. These results are in line with a previous observation that optogenetic activation of Type III cells decreased CT nerve responses to sucrose [50]. On the other hand, inhibition of sour responses by sweetness is unlikely because prior optogenetic studies have shown that sweet stimuli did not affect CT nerve responses elicited by light stimulation of Type III (sour-sensitive) taste cells [50]. To further elucidate the impact of the absence of GABA in Type III cells on sweet taste responses, additional studies, such as single-fiber recordings of sweet fibers in *Gad67*-cKO mice, are required. Taken together, our results suggest that the suppression of sweet tastes by sour may occur in part due to peripheral inhibition of Type II, sweet responsive taste cells by GABA released by sour-responsive Type III cells. In the case of saccharin, *Gad67*-cKO mice still exhibited some inhibition when stimulated with a sour–sweet mixture (Fig. 6). One possible explanation for this exception could be that the sweet–sour interaction is mediated at the receptor level (TAS1R2 or TAS1R3) by ligand binding. Acidity might affect the receptor-ligand binding between saccharin and TAS1R2 or TAS1R3, similar to the binding of miraculin and sweet receptors. This possibility should also be investigated in future studies.

In this study, we focused on responses to sweet–sour mixtures to examine the effect of GABA in Type III cells on peripheral taste interaction. However, there is a possibility that GABA also affects responses to bitter and/or umami taste cells, which are all Type II taste cells. Indeed, Vandenbeuch et al. (2020) showed that optogenetic activation of Type III cells reduced bitter (quinine) responses [50], and Dvoryanchikov et al. (2011) demonstrated that GABA reduced ATP release elicited by stimulation of a sweet-bitter mixture [12]. Because of the small responses to bitter and umami compounds in the CT nerve, we have not investigated this possibility in this study. For a comprehensive understanding of the role of GABA in Type III cells, future studies are necessary to investigate the bitter-sour and umami-sour interactions using *Gad67*-cKO mice.

GABA released from Type III cells could activate GABA receptors on sweet-sensitive taste cells. A previous study demonstrated that Type II cells express both GABA_A_ and GABA_B_ receptors, and that both muscimol (a GABA_A_ agonist) and baclofen (a GABA_B_ agonist) reduced ATP release from Type II cells [13]. This suggests a possibility that both GABA_A_ and GABA_B_ receptors are involved in the suppression of sweet responses by sour taste. In this study, we used anesthetics including midazolam, which is known to enhance the effect of GABA [27, 52]. Therefore, the sour-induced sweet inhibition observed in the CT nerve responses of WT mice in this study might be stronger than under normal conditions. It is plausible that sour-induced sweet inhibition in non-anesthetized animals may be weaker than what was observed in this study. In addition, other mechanisms may also explain our observations. In neurons of the dorsal root ganglion, the GABA depolarization seems to desensitize the primary afferent terminals [44]. If a similar mechanism occurs in taste buds, GABA release could potentially cause a slight depolarization of primary gustatory fibers, making them less responsive to ATP released by Type II cells. Acidification of the epithelium may also affect function of the GABA_A_ receptors, as protons have been shown to inhibit GABA-activated currents in rat primary sensory neurons [61]. Future studies will elucidate the mechanisms by which sour stimuli inhibit sweet taste at the peripheral level.

Taste interactions occur whenever we eat something, but much remains unknown about their specific physiological functions. In this study, we demonstrated that sour-induced sweet inhibition occurs at the taste bud level. However, previous studies on taste interactions have shown that these interactions may occur at different levels and through various mechanisms. For example, the suppression of sweetness by bitterness has been reported at the level of taste receptors [16, 18, 24], synapses of peripheral neurons [10], and central neural circuits [25]. These interactions all result in the reduction of the sweet signal, which is crucial for eliciting a preferable sensation. Food intake in animals may depend on the balance between preferable and aversive signals. Thus, the reduction of preferable signals could lead animals to avoid certain foods. Aversive signals, such as bitter and sour tastes, are thought to play a defensive role. Therefore, the reduction of preferable signals by adding aversive compounds may be important for preventing the ingestion of harmful substances, thereby protecting the body.

## Data Availability

No datasets were generated or analysed during the current study.
